# The effect of absorbable collagen suture for oral implant repair on wound healing and inflammation factors of gingival crevicular fluid

**DOI:** 10.5937/jomb0-51148

**Published:** 2025-01-24

**Authors:** Feng Jingjing, Yu Qiaolong, Liu Xiaoqing

**Affiliations:** 1 Fujian Medical University Affiliated Sanming First Hospital, Department of Stomatology, Sanming, China; 2 The 73rd Army Hospital of Chinese PLA, Department of Stomatology, Xiamen, China; 3 Fujian Medical University, School of Stomatology, Fuzhou, China + Xiamen University, School of Medicine, Department of Stomatology, Xiamen, China; 4 The Third Clinical Medical College of China Three Gorges University Gezhouba Central Hospital of Sinopharm Yichang 443002, Stomatology Center, Hubei, China

**Keywords:** absorbable, collagen suture, implant repair, gingival crevicular fluid, inflammatory factors, apsorbujući, kolagenski šav, popravka implantata, gingivalna pukotina, inflamatorni faktori

## Abstract

**Background:**

To investigate the clinical value of absorbable collagen suture in the treatment of oral implant restoration.

**Methods:**

A prospective, randomized, single-blind trial was conducted in patients undergoing dental implant restoration in our hospital. The patients were divided into an absorbable group (incision closure with absorbable collagen suture) and a conventional group (incision closure with conventional suture). The incision healing time, postoperative pain degree, incision healing grade, patient satisfaction, and the levels of tumor necrosis factor-a (TNF-a), interleukin-8 (IL-8) and interleukin-6 (IL-6) in gingival crevicular fluid were compared between the two groups.

**Results:**

The absorbable group had faster incision healing times and lower postoperative pain scores on days 1 and 2, all with significant differences (P<0.05). Wound healing in the absorbable group was notably better, with a Grade A healing rate of 96.88% and a Grade B healing rate of 3.13%. In contrast, the conventional group had a Grade A healing rate of 81.25%, a Grade B healing rate of 17.19%, and a Grade C healing rate of 1.56%. These differences favored the absorbable group significantly (P<0.05). Before surgery, there were no statistically significant differences in the levels of TNF-a, IL-6, and IL-8 in gingival crevicular fluid between the absorbable and conventional groups (P>0.05). However, 3 days after surgery, the absorbable group showed significantly lower levels of TNFa, IL-6, and IL-8 compared to the conventional group (P<0.05). Patient satisfaction rates for stability, aesthetics, chewing function, and pronunciation were similar between the two groups (P>0.05). However, patients in the absorbable group reported significantly higher comfort levels compared to those in the conventional group (P<0.05). Moreover, the complication rate in the absorbable group was significantly lower at 6.25% compared to 18.75% in the conventional group (P<0.05).

**Conclusions:**

Absorbable collagen suture for oral implant prosthesis after suture, beneficial to wound healing and reduce postoperative pain and inflammation.

## Abstract (sr)

**Uvod:**

Cilj je bio da se istraži klinička vrednost upijajučeg kolagenskog šava u lečenju restauracije oralnih implantata.

**Metode:**

Prospektivno, randomizovano, jedno-slepo ispitivanje sprovedeno je kod pacijenata koji su bili podvrgnuti restauraciji zubnih implantata u našoj bolnici. Pacijenti su podeljeni u apsorbujuču grupu (zatvaranje rezom sa upijajučim kolagenskim šavom) i konvencionalnu grupu (zatvaranje incizije konvencionalnim šavom). Vreme zarastanja incizije, postoperativni stepen bola, stepen zarastanja incizije, zadovoljstvo pacijenata i nivoi faktora tumorske nekroze-a (TNF-a), interleukina-8 (IL-8) i interleukina-6 (IL-6) u gingivi crevikularna tečnost upoređena između dve grupe.

**Rezultati:**

Grupa koja se apsorbuje imala je brže vreme zarastanja rezova i niže rezultate postoperativnog bola 1. i 2. dana, sve sa značajnim razlikama (P<0,05). Zarastanje rana u apsorbovanoj grupi bilo je znatno bolje, sa stopom zarastanja stepena A od 96,88% i stopom zarastanja stepena B od 3,13%. Nasuprot tome, konvencionalna grupa je imala stopu zarastanja stepena A od 81,25%, stopu zarastanja stepena B od 17,19% i stopu zarastanja stepena C od 1,56%. Ove razlike su značajno favorizovale apsorpcionu grupu (P<0,05). Pre operacije, nije bilo statistički crevikularnoj tečnosti između apsorbujuče i konvencionalne grupe (P >0,05). Međutim, 3 dana nakon operacije, apsorbovana grupa je pokazala značajno niže nivoe TNF-a, IL-6 i IL-8 u poređenju sa konvencionalnom grupom (P<0,05). Stope zadovoljstva pacijenata za stabilnost, estetiku, funkciju žvakanja i izgovor je bio sličan između dve grupe (P>0,05). Međutim, pacijenti u apsorpcionoj grupi su prijavili značajno više nivoe udobnosti u poređenju sa onima u konvencionalnoj grupi (P<0,05). Staviše, stopa komplikacija u apsorbovanoj grupi bila je značajno niža od 6,25% u poređenju sa 18,75% u konvencionalnoj grupi (P<0,05).

**Zaključak:**

Upijajuči kolagenski šav za oralne implantate proteze nakon šava, koristan za zarastanje rana i smanjenje postoperativnog bola i upale.

## Introduction

Oral implantology is a relatively new branch of stomatology with a relatively short history. In recent years, with the continuous improvement of modern people’s awareness of oral health, more and more patients choose oral implant surgery to repair the problem of missing teeth. The core concept of dental implant surgery is to create artificial roots by using non-biological or biological materials, and then implant these artificial roots into the alveolar bone of the patient to replace the missing tooth. Compared with traditional denture (base), oral implants can significantly improve the comfort and quality of life of patients. This is because oral implant teeth do not need to rely on the surrounding teeth as support, but rely on the implanted artificial root, and therefore are more stable and have excellent retention effect. As an important development in the field of stomatology, oral implant medicine provides a reliable solution to improve the oral health and quality of life of patients who have lost their teeth. Recent studies have found that the use of different types of prostheses may lead to a series of stress responses in patients who need to restore missing dentition [1]. These include the patient’s physiological and biochemical reaction, often happen after treated with oral cavity repair. These stress may trigger the body to release inflammatory factors and other biochemical signals, lead to local inflammation. This kind of situation is often viewed as part of the normal healing process, but a different type of restoration may be different influence on the reaction. Some prosthetic materials may be more suitable for compatibility with human tissues, helping to reduce the inflammatory response and promote healing. It has been confirmed that the use of different types of suture materials may trigger different degrees of severe reactions during the healing process of oral mucosa. Among them, collagen threads are considered to have better histocompatibility and help to promote the wound healing process. However, despite this, there is still an inconsistency regarding which type of suture to use in clinical practice. The problem requires more research and clinical trials, in order to make clear the applicability of different suture materials, and their ease of stress reaction and promoting healing in patients. This will help the field of stomatology to better select appropriate suture materials to provide a more effective and comfortable treatment regimen while reducing unnecessary inflammatory responses [2]. This study observed the effects of two kinds of sutures in oral implant restoration, in order to provide guidance and basis for clinical practice.

## Materials and methods

### Study design

A total of 128 patients undergoing implant restoration in our hospital were enrolled in this prospective, randomized, single-blind trial. The patients were divided into the absorbable group (the incision was sutured with absorbable collagen suture) and the conventional group (the incision was sutured with conventional suture), with 64 cases in each group. Inclusion criteria: (1) patients required implant restoration; (2) All single maxillary and mandibular implant restorations; (3) 18 ~ 59 patients age range; (4) The patients had good oral hygiene with 0-i degree calculus: (5) the gingival thickness was 1.0-3.0 mm; (6) Informed consent was obtained from patients before treatment. Exclusion criteria: (1) Oral combined with facial deformity or tumor; (2) serious function of liver and kidney disease; (3) chronic periodontal inflammation and oral infectious diseases; (4) The alveolar ridge needs a lot of trimming, guided bone regeneration and gingival removal. (5) Alcoholism. Absorbable group, aged from 18 to 59 years, mean 41.3±7.6 years; There were 45 males and 19 females. Plant area: maxillary 34 cases, 30 cases of mandibular; The mean gingival thickness was 1.54±0.38 mm (range, 1.04–2.84 mm). Plaque: 0 degrees 44 cases, I 20 cases. The conventional group, aged from 18 to 59 years, with an average of 40.5±7.8 years; There were 48 males and 16 females. There were 30 maxillary implants and 34 mandibular implants. Gingival thickness 1.08 ~ 2.67 mm, the average thickness of gum 1.48 + / - 0.32 mm; Plaque: 0 degrees of 48 cases, I 16 cases. There was no significant difference in age, gender and other baseline data between the absorbable group and the conventional group (P>0.05).

In both groups, oral implantation was carried out, the oral and perioral skin was disinfected, 2% lidocaine anesthesia was used to make an curved incision on the alveolar crest, the periosteum and mucosa were cut, and the bone surface was exposed. The positioning guide plate was used to locate the hole. The first stage split drill was used to expand the hole, and the second stage split drill was used to expand the whole process. Implanted at the top of the screw, the washing and suture wounds. In the conventional group, the incision was sutured with conventional suture, the tension free suture was carried out with silk weaving without the risk of absorption, the aseptic operation was strictly performed, and the anti-inflammatory treatment was given for 3 days after operation. In the absorbable group, the incision was sutured with absorbable collagen suture, and the wound was closed by simple intermittent suture with absorbable collagen suture. Two to four stitches were sutured according to the alveolar condition, and three knots were tied at each point using a needle holding device.

The incision healing time and postoperative pain degree of patients were compared between the two groups (the pain degree of patients was evaluated by visual analogue scale (VAS), with a full score of 10 and a minimum score of 0). The higher the score, the more severe the pain was), the grade of incision healing (divided into grade A, B and C), and the levels of tumor necrosis factor-a (TNF-α), interleukin-6 (IL-6) and interleukin-8 (IL-8) in gingival crevicular fluid. The gingival crebular fluid (GCF) was collected and dried. The surface of the gingiva, implant abutment and denture crown or natural crown was wiped with a sterile cotton ball, and the filter paper was gently inserted into the gingival sulcus around the implant or natural teeth until slight resistance was felt. The levels of tumor necrosis factor-a (TNF-α), interleukin-6 (IL-6), and interleukin-8 (IL-8) in gingival crevicular fluid were measured by enzyme-linked immunosorbent assay.

Patient satisfaction survey, mainly investigate patients for dental implant stability, aesthetic, chewing function, pronunciation, comfort, satisfaction, the highest was divided into 10 points, out of ten, 7–10 - for satisfaction, 5–6 is divided into general, four points and the following is not satisfied.

This study was approved by the ethics committee. Signed written informed consents were obtained from the patients and/or guardians.

### Statistical analysis

The statistical analysis was performed using Statistic Package for Social Science (SPSS) 21.0 software (IBM, Armonk, NY, USA). Quantitative indicators, including the levels of TNF-α, IL-6, and IL-8 in gingival crevicular fluid for both groups, were represented as mean (± standard deviation). Group comparisons for the above-mentioned quantitative indicators were conducted using the t-test. Patient satisfaction survey data and gender distribution fell under the category of categorical variables. Comparative analyses between the two groups for these variables were carried out using the chi-square test or non-parametric tests. A significance level of P<0.05 was considered as statistically significant differences.

## Results

The incision healing time of the absorbable group was shorter than that of the conventionalgroup, and the difference was statistically significant (P<0.05). Compared with the pain severity of the two groups at 1 day and 2 days after operation, the VAS score of the absorbable collagen suture group was lower than that of the conventional group (P<0.05), indicating that the absorbable collagen suture was beneficial to reduce the pain of the patients during wound healing at the early stage. Refer to Table 1.

**Table 1:** Comparison of Incision Healing Time and VAS Scores in Two Patient Groups (±SD).

Group	n	Incision healing time (d)	VAS score		
			Postoperation 1d	Postoperation 2d	Postoperation 3d
the absorbable group	64	5.56±0.94	3.30±0.87	1.96±0.62	0.83±0.36
the conventional group	64	7.02±1.13	3.87±0.92	2.26±0.68	0.95±0.41
t		-7.946	-3.601	-2.608	-1.759
P		0.000	0.000	0.010	0.081

The healing grade of surgical incision in the two groups was observed. The healing rates of grade. A incision in the absorbable group and the conventional group were 96.88% and 81.25%, respectively, the healing rates of grade B incision were 3.13% and 17.19%, respectively, and the healing rate of grade C incision in the conventional group was 1.56%. The wound healing effect of the absorbable group was significantly better than that of the conventional group (P<0.05). Refer to Table 2.

**Table 2:** Comparison of wound healing grades between the two groups (n (%)).

Group	n	Class A	Class B	Class C
the absorbable group	64	62(96.88)	2(3.13)	0(0.00)
the conventional group	64	52(81.25)	11(17.19)	1(1.56)
Z		-2.828		
P		0.005		

The levels of inflammatory cytokines (TNF-α, IL-6, IL-8) in the gingival crevicular fluid of the two groups before surgery were detected. There was no significant difference in the degree of inflammatory response between the two groups before surgery (P>0.05). Three days after surgery, the degree of inflammatory reaction in the absorbable group was lighter, and the levels of TNF-α, IL-6 and IL-8 in the gingival crevicular fluid were significantly lower than those in the conventional group (P<0.05). Refer to Table 3.

**Table 3:** Comparison of TNF-α, IL-6 and IL-8 levels in gingival crevicular fluid between the two groups before and after surgery (±s).

Group	n	TNF-α (mg/L)		IL-6 (μg/L)		IL-8 (μg/L)	
		Preoperative	Postoperation 3d	Preoperative	Postoperation 3d	Preoperative	Postoperation 3d
the absorbable<br>group	64	2.25±0.89	4.17±1.05	15.42±3.76	28.94±6.22	39.85±8.42	75.20±12.33
the conventional<br>group	64	2.10±0.73	4.83±1.12	16.50±4.16	38.00±8.19	41.32±9.06	89.56±15.13
t		1.042	-3.439	-1.541	-7.048	-0.951	-5.886
P		0.299	0.001	0.126	0.000	0.344	0.000

After operation, the complication rate of the absorbable group was 6.25%, which was lower than 18.75% of the conventional group, and the difference was statistically significant (P<0.05). Refer to Table 4.

**Table 4:** Comparison of complication rates between the two groups.

Group	n	The gums oozed blood	Redness and swelling of the gum	Complication rate (%)
the absorbable group	64	1	3	4(6.25)
the conventional group	64	5	7	12(18.75)
χ^2^				4.571
P				0.033

After the operation, the satisfaction of the two groups was investigated. There was no significant difference in the stability, aesthetics, masticatory function, and pronunciation satisfaction between the absorbable group and the conventional group (P>0.05). Can absorb the comfort of a group of patients satisfaction in higher than normal group (P < 0.05); Refer to Table 5.

**Table 5:** Patient Satisfaction Survey in Two Groups (n (%)).

Group	n	stability	aesthetic<br>degree	masticatory<br>function	pronounce	comfort level
the absorbable group	64	62(96.88)	60(93.75)	61(95.31)	60(93.75)	61(95.31)
the conventional group	64	58(90.63)	55(85.94)	55(85.94)	56(87.5)	53(82.81)
χ^2^		2.133	2.14	3.31	1.471	5.133
P		0.144	0.143	0.069	0.225	0.023

## Discussion

Implant denture is a common treatment method in clinical dentistry, which has the advantages of wide indications and good cosmetic effects, and is widely used in denture restoration. With the development of modern dental materials and surface treatment technology, the success rate of dental implants has been greatly improved [3]. In recent years, reports have pointed out that although planting denture retention is higher, but the price is expensive, cycle is long so problems once appear, implant will give patients’ physical and mental damage, particularly around the implant infection happened belongs to chronic progressive inflammatory disease, refers to the mucous membrane and alveolar bone around implant showed symptoms of inflammation, leading to loss of alveolar bone around implant, It can cause implant loosening and eventually lead to implant failure [4]. Studies have shown that the periodontal ligament is a connective tissue that wraps around the root of the tooth and is composed of a variety of cells such as fibroblasts, osteoblasts, and osteoclasts. When bacteria invade the periodontium, they cause a strong, nonspecific response. In the gingival pocket and periodontal pocket of patients, a large number of bacteria, mainly anaerobic bacteria, usually accumulate. These bacterial metabolites and endotoxins etc. can lead to the production of large amounts of free radicals. The occurrence of periodontitis is largely due to the formation of free radicals and the clearance between the balance is broken [5] [6]. In recent years, with the development of oral medicine planting techniqueof suture, and suture material appear larger improvements, in plant operation, the main purpose of the suture is keep the gum tissue in the appropriate position, until it fully recovered. This helps to prevent bone tissue from being exposed to soft tissue, while also having the effect of stopping bleeding and eliminating postoperative cavities. In addition, suturing helps to reduce postoperative gingival pain and make the gums better return to their normal position [7]. Traditional suture materials will produce obvious scars in the patient’s gingiva, which will affect the formation after termination in severe cases. Animal experiments have also found that different suture materials will affect the healing of oral mucosa. In addition, the traditional risk is that soft scale is easy to attach to the suture tip, leading to bacterial accumulation and affecting incision healing [8].

This study observed the absorbable collagen suture application in the oral cavity planting treatment, absorbable sutures collagen because of its overall absorption properties, hindered the adhesion of plaque and bacteria. This is very beneficial for maintaining oral hygiene and reducing the accumulation of inflammation, thus effectively reducing the possibility of postoperative infection [9]. Absorbable collagen sutures have stable chemical properties, good compatibility with organisms, and do not cause obvious tissue reactions. Some experts believe the material helps improve the healing process of wounds after surgery. It can be accepted by the body in a short time and absorb, generally in 5 to 8 days. Within this time frame, the oral mucosa is allowed to undergo natural regeneration and repair [10]. Studies have shown that oral tissues are able to uniformly absorb absorbable collagen sutures, degrading them to protein. This helps to avoid rejection, and promote the local blood coagulation process, strengthen the hemostatic effect. In addition, this suture can also maintain the smoothness and some elasticity of the oral tissue [11]. Absorbable suture collagen degradation process match the wound healing step, help stabilize the oral tissue, and to provide structural support for the recovery of wounds, which will help to shorten the healing time. Suture use aldehyde crosslinking technology can slow the degradation rate of collagen. After oral surgery to repair, the mouth will significantly influence on the environment and occlusion of the collagen suture absorption process [12]. This study analyzed the changes of a variety of inflammatory factors in the patient’s body. TNF-α is synthesized and secreted by macrophages and monocytes, which initiates the inflammatory response of the body, causes the enhancement of osteoclast activity, and causes connective tissue damage, which affects the repair of periodontal tissue [13] [14] [15]. IL-6 can stimulate the release of inflammatory factors, resulting in systemic inflammatory response, which is also an indicator of high sensitivity and specificity. IL-8 is a multi-source inflammatory factor, which enhances the chemotactic effect of neutrophils and T cells, leading to the accumulation of inflammatory factors and different degrees of tissue damage. Therefore, the use of absorbable collagen suture will produce less stimulation of periodontal tissue, reduce the formation of irritants, and protect the function of teeth [16]. On the one hand, we should actively treat and control patients with periodontal disease to reduce the presence of peri-implant inflammation. On the other hand, the affected teeth that affect the implant restoration should be extracted, especially the affected teeth that may seriously affect the appearance of patients and the implementation of long-term restoration programs should be removed as soon as possible to improve the oral function and aesthetics of patients after implantation [17] [18] [19] [20]. At the same time to strengthen health education, to contribute to the success of planting risk factors include excessive smoking, chewing habits to be correct, keep patients after planting good oral status.

This study showed that can absorb a group of patients with incision healing time is short in the conventional group, postoperative 1 d and 2 d postoperatively, patients can be absorbed VAS score lower than normal group, suggests adopting absorbable collagen suture for oral implant prosthesis after suture can shorten the incision healing time, reduce the degree of pain after treatment. In the absorbable group, the healing rate of grade A incision was 96.88%, and the healing rate of grade B incision was 3.13%. In the conventional group, the healing rate of grade A incision was 81.25%, the healing rate of grade B incision was 17.19%, and the healing rate of grade C incision was 1.56%. Three days after surgery, the levels of TNF-α, IL-6, and IL-8 in gingival crevicular fluid of the absorbable collagen suture group were lower than those of the conventional group, suggesting that the use of absorbable collagen suture for oral implant repair is beneficial to reduce the degree of inflammatory response. After surgery, can absorb group of patients with complication rate was 6.25% less than 18.75% of the conventional group of patients, suggests adopting absorbable collagen suture for oral implant prosthesis after suture can reduce postoperative complications. The results showed that the comfort satisfaction of the absorbable collagen suture group was higher than that of the conventional group (P<0.05), which indicated that the absorbable collagen suture was effective in maintaining the function of implant restorationand had obvious advantages in improving the comfort of patients. Analysis of the reason for the material of absorbable collagen suture and structure to make it more soft, more adapt to the organization, reduces the surrounding soft tissue irritation and discomfort. Second, since is absorbable sutures, patients do not need to see a doctor again to remove stitches, this greatly reduces the number of visits and the related inconvenience. In addition, absorbable sutures cause less inflammatory response, which helps to reduce postoperative discomfort and pain, thereby improving patient comfort. Although the two sutures have similar effects in terms of stability, aesthetics, masticatory function and pronunciation, the good suture effect of absorbable suture is more helpful to improve the overall satisfaction of patients. Finally, this suture material promotes a faster healing process and reduces the risk of complications, factors that combine to indirectly improve patient comfort.

This study compares the two kinds of sutures used in the role of oral planting, confirmed the effectof absorbable collagen suture is better, laid the foundation for clinical reasonable select sutures, but because the research time is short, less number into the group of patients, follow-up time is short, so still need to extend the sample sizes, longer follow-up time in-depth analysis. Future studies will involve a longer follow-up period to assess the long-term outcomes of using absorbable collagen sutures. Additionally, a larger sample size will be considered to enhance the generalizability of the findings. More comprehensive measures of patient-reported outcomes and objective clinical assessments will be included to provide a holistic view of the benefits and potential drawbacks of absorbable sutures in oral implantology.

In conclusion, the use of absorbable collagen sutures in the suture of oral implant restoration is beneficial to wound healing, postoperative pain and inflammatory response.

## Dodatak

### Conflict of interest statement

All the authors declare that they have no conflict of interest in this work.

### Contribution

Jingjing Feng and Qiaolong Yu contributed equally to this work.
